# Comment on: “exercise training and cardiac autonomic function following coronary artery bypass grafting: a systematic review and meta-analysis”

**DOI:** 10.1186/s43044-023-00344-7

**Published:** 2023-03-16

**Authors:** Caroline Oliveira Gois, Lino Sergio Rocha Conceição, Alana Lalucha de Andrade Guimarães, Vitor Oliveira Carvalho

**Affiliations:** 1 Postgraduate Program in Health Science, Department of Physical Therapy, Federal University of Sergipe (Universidade Federal de Sergipe - UFS), Av. Marechal Rondon, s/n -Jardim Rosa Elze, São Cristóvão, Sergipe 49100-000 Brazil; 2 The GrEAt Group (Grupo de Estudos Em Atividade Físicasica), Av. Marechal Rondon, s/n -Jardim Rosa Elze, São Cristóvão, Sergipe 49100-000 Brazil

**Keywords:** Exercise training, Coronary artery bypass graft, Heart rate variability, Heart rate recovery, Cardiorespiratory fitness

## Abstract

**Background:**

Low cardiorespiratory fitness is associated with poor prognosis in individuals with coronary artery disease and after coronary artery bypass grafting surgery. Thus, we comment about a meta-analysis that adds important information about the effect of exercise training on cardiac autonomic function in individuals following coronary artery bypass grafting surgery.

**Main body:**

The study by Kushwaha et al. showed positive effects for heart rate variability and heart rate recovery in subjects after coronary artery bypass grafting surgery in response to acute physical training. These data are relevant, since heart rate variability is an independent predictor of for all-cause and cardiovascular mortality for individuals with cardiovascular disorders. Additionally, attenuated heart rate recovery is associated with increased risk for the same outcomes. Moreover, we summarize the quantitative data from studies that compared the effect of physical training in comparison with control group in cardiorespiratory fitness in adults following coronary artery bypass grafting.

**Conclusions:**

Our findings suggest that improvements in peak oxygen consumption result in an additional benefit in adults following coronary artery bypass grafting. Considered that, the increased cardiorespiratory fitness is an independent predictor of longer survival in coronary artery disease.

## Background

We read with great interest the systematic review by Kushwaha et al. [1] that adds important information on the effects of exercise training on cardiac autonomic function in individuals after coronary artery bypass grafting (CABG) surgery. The study shows positive effects for heart rate variability (HRV) and heart rate recovery (HRR) in subjects after CABG surgery in response to acute physical training [1].

In their results, the authors reported that, for HRV in the time domain, there was an increase in the standard deviation of RR intervals (SDNN) (SMD 0.44 [0.17–0.71], *P* = 0.002, *I*^2^ 0%) and increase in square root of the mean squared differences between successive RR intervals (RMSSD) (SMD 0.68 [0.28–1.08], *P* = 0.0008, *I*^2^ 7%). Furthermore, results showed an increase in high frequency (HF) domain (SMD 0.58 [0.18–0.98], *P* = 0.0005, *I*^2^ 35%), which may reflect additional positive changes in parasympathetic tone. In addition, suggesting an overall balance of sympathetic and parasympathetic function, there was a reduction in the low frequency-to-high frequency ratio (LF/HF) (SMD − 0.34 [ − 0.65 to 0.02], *P* = 0.03,* I*^2^ 0%). Moreover, for HRR in the first minute after exercise, significant improvement was demonstrated (SMD 0.71 [0.39–1.02], *P* < 0.001, *I*^2^ 0%), corresponding to a larger decrease HRR, that is, a greater reactivation of the parasympathetic nervous system [1, 2]. These data are relevant, since regulation of the cardiac autonomic nervous system is an important outcome of physical training [2].

## Main text

Conceptually, HRV and HRR are indirect, noninvasive, reliable and safe measures for monitoring and assessing cardiac autonomic control. HRV is an independent predictor of all-cause and cardiovascular mortality for individuals with atrial fibrillation [3]. In addition, low HRV is associated with a higher risk of heart failure with preserved ejection fraction and with a higher incidence of hospitalization for heart failure in postmenopausal women [4]. Attenuated HRR is associated with increased risk of cardiovascular events and all-cause mortality [5] (Table 1).

**Table 1:** Cardiac autonomic function variables significantly predictive or associative of relevant clinical outcomes

Clinical conditions	HRV	Time domain	Frequency domain
Hemodialysis [6]	All-cause mortality: decreased HRV (HR 1.63 (95% CI 1.11–2.39), *P* = 0.014, *I*^2^ = 74.2%) (association)*	All-cause mortality: decreased SDANN (HR 1.03 (95% CI 1.01–1.05), *P* = 0.001, *I*^2^ = 21.8%)	All-cause mortality: decreased LF/HF ratio (HR 8.69 (95% CI 2.24–33.68), *P* = 0.002, *I*^2^ = 53.5%)
	CV mortality: decreased HRV (HR 1.07 (95% CI: 1.00–1.15), *P* = 0.045, *I*^2^ = 89.7%) (association)*		
Cancer (pancreatic cancer, breast cancer, advanced non-small cell lung cancer and mixed cancer types) [7]	Overall survival: between the higher HRV group and the lower HRV group (HR 0.70 (95% CI 0.60–0.82), *P* < 0.001, *I*^2^ = 27%)		
Unstable angina and preserved left ventricular function [8]		In‐hospital death: SDNNi < 39 ms (OR 4.99 (95% CI 1.18–21.1), *P* = 0.029)	In‐hospital death: LF < 15,7 ms (OR 4,94 (95% CI 1,16–20,9), *P* = 0,030); LF/HF ratio < 1.12 (OR 5.14 (95% CI 1.21–21.8), *P* = 0.026)
Congestive heart failure [9]			Cardiac events: VLF In (ms2) 6.24, (Chi-square), *P* = 0.01)
Severe sepsis [10]		Survivors: SDNN value was significantly higher among survivors (SD 18.50 (IR 10.00–34.50)) as compared to non-survivors (SD 8.50 (IR 5.00–14.50), *P* = 0.003). SDNN (ms) (HR 0.937 (95% CI 0.883–0.995), *P* = 0.033)	
Finnish adult population [11]	Total mortality: HRV morning-evening (RH 1.08 (95% CI 1.03–1.13), *P* < 0.001); HRV morning day-by-day (RH 1.11 (95% CI 1.05–1.17), *P* < 0.001) Fatal and nonfatal CV events: HRV morning day-by-day (RH 1.11 (95% CI 1.05–1.17), *P* < 0.001)		

*Decreased HRV was associated with higher all-cause mortality and cardiovascular mortality

*HRV* Heart rate variability, *VLF* very low frequency, *CV* cardiovascular, *SDNNi* standard deviations of RR intervals of all 5 min segments, *HF* high frequency, *SDANN* standard deviations of RR intervals of all 5 min segments, *LF* low frequency

Physical training is a Class I recommendation and one of the main elements in the cardiac rehabilitation program by the American Heart Association College of Cardiology and the European Society of Cardiology. Physical training increases cardiorespiratory fitness, reduces the risk of cardiovascular mortality, acute myocardial infarction and hospitalization [12]. In addition, a meta-analysis showed that resistance training resulted in improvement of all HRV parameters in the time and frequency domains, in contrast to resistance training and aerobic high-intensity interval combined. A meta-regression also showed that after the physical training program, improvement in LF/HF domains was significantly associated with improvement in peak oxygen consumption (VO2 peak) [coefficient:  − 0.05 ( − 0.081 to 0.019), *P* = 0.005, *I*^2^ 0.0%] [13]. In conclusion, studies show that physical training alone or a combination of aerobic and resistance training leads to adaptations in cardiac autonomic control (Table 2).

**Table 2:** Effect of physical training on heart rate variability

Conditions	Modality	HRV—time and frequency domains
Older people [14]	Training protocols were subdivided according to their frequency and duration	SDNN 0.721 (0.184–1.257), *P* = 0.008, *I*^2^ 41.72%
Hypertensive women [15]	Aerobic and resistance training	SDNN + 54.3%, *P* < 0.001; HFnu + 66.8%, *P* < 0.001; RMSSD + 37.3%, *P* < 0.001; LF/HF − 68.6%, *P* < 0.001
Type 2 diabetes mellitus [13]	General training	SDNN 0.59 (0.26–0.93), *I*^2^ 69.5%; RMSSD 0.62 (0.28–0.95), *I*^2^ 60.8%; LF/HF − 0.52 ( − 0.79 to − 0.24), *I*^2^ 61.1%; LF -0.37 ( − 0.69 to − 0.05),* I*^2^ 78.9%
	Endurance	SDNN 0.65 (0.19–1.10), *I*^2^ 68.4%; RMSSD 0.66 (0.21–1.11), *I*^2^ 66.5%; LF/HF − 0.49 ( − 0.74 to − 0.24), *I*^2^ 18.4%; LF − 0.55 ( − 0.95 to − 0.15), *I*^2^ 79.6%; HF − 0.56 (0.18–0.94), *I*^2^ 72.8%
	Resistance	LF − 0.93 ( − 1.56 to − 0.3), *I*^2^ 0%; LF/HF − 0.96 ( − 1.59 to − 0.33), *I*^2^ 0%
	Endurance combined with resistance	–
	High-intensity interval training	RMSSD 1.26 (0.78 – 1.94) *I*^2^ -; LF/HF − 1.63 ( − 2.64 to − 0.62), *I*^2^ 65.2%

*HRV* Heart rate variability, *RMSSD* Square root of the mean squared differences between adjacent RR intervals, *LF* Low frequency, *HFnu* High frequency normalize units, *LF/HF* Ratio of low and high frequency, *SDNN* Standard deviation of the RR intervals

Previous studies have shown that there are differences in the magnitude of changes in HRV induced by physical training according to training protocol [2]. In the study by Kushwaha et al. [1], an interesting point was the inclusion of studies that analyzed patients with and without beta-blocker therapy (5 [16–20] and 3 [21–23] studies respectively). Nevertheless, the study reported improvements in HRV and HHR. Beta-blockers are the first-line therapy to control symptoms in stable coronary artery disease and to reduce exercise-induced angina [24, 25]. Beta-blockers modify heart rate [26] and, thus, can affect HRV and HRR [25]. Therefore, as a contribution to the present study, we suggest that further clinical trials and systematic reviews should consider this type of analysis to see how it affects the magnitude of the effect and heterogeneity.

**Fig. 1:**
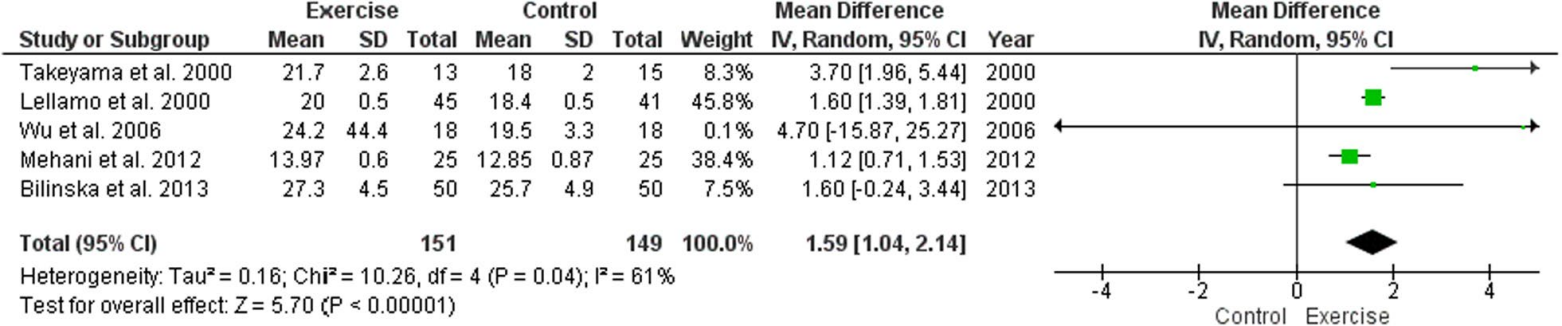
Forest plot of comparison: exercise versus control, outcome: peak oxygen consumption

In addition to these findings, other issues regarding to the cardiorespiratory fitness of individuals after CABG surgery need to be considered. In 2016, a scientific statement from the American Heart Association considered the importance of assessing cardiorespiratory fitness in clinical practice considering it as a clinical vital sign [27]. Moreover, it is already well described in the scientific literature that low cardiorespiratory fitness is associated with poor prognosis in individuals with coronary artery disease and after CABG surgery [24, 27]. Despite the excellent systematic review with meta-analysis performed by Kushwaha et al. [1], no meta-analysis has been performed for VO2 peak. When pooling the available studies for the meta-analysis comparing physical training (different types of exercise training including aerobic, resistance, interval and combined aerobic and resistance training either alone or combined) with controls (no exercise), we observed significant effect for the VO2 peak in the physical training group (MD = 1.59 mL O_2_ Kg^−1^ min^−1^, (95% CI 1.04–2.14, *I*^2^ = 61%, 5 [16, 17, 19, 23, 28] studies, *N* = 300, *P* < 0.00001), (Fig. 1). Considering the results presented by Kushwaha et al. [1] and confirmed by our meta-analysis, the improvement in HRR can also be associated with improvement in cardiorespiratory fitness [1, 27]. Improvements in cardiorespiratory fitness play a role as an independent predictor for survival in coronary artery disease. In addition, increments in VO2 peak have been associated with a 14–17% reduction in the risk of cardiovascular disease and death from all causes [24]. Therefore, it is reasonable for healthcare professionals to assess the cardiorespiratory fitness of individuals after CABG.

## Conclusions

In conclusion, we congratulate Kushwaha et al. [1] in this important research. Additionally, we provide information about cardiorespiratory fitness of the included studies that showed a difference of 1.59 mL O_2_ Kg^−1^ min^−1^ in VO2 peak between physical training and control after CABG surgery. Finally, it is important to address the need for further studies to investigate if there is any association between improvements in cardiorespiratory fitness and improvement cardiac autonomic function (HRV and HRR), as well as additional benefits from physical/exercise training in adults after CABG surgery.

## Data Availability

Not applicable.

## Abbreviations

CABG: Coronary artery bypass grafting
HRV: Heart rate variability
HRR: Heart rate recovery
SDNN: Deviation of RR intervals
RMSSD: Squared differences between successive RR intervals
HF: High frequency
LF/HF: Low frequency-to-high frequency ratio
VO2 peak: Peak oxygen consumption
VLF: Very low frequency
CV: Cardiovascular
SDNNi: Standard deviations of RR intervals of all 5 min segments
SDANN: Standard deviations of RR intervals of all 5 min segments
LF: Low frequency
HFnu: High frequency normalize units
